# Long-term evaluation of uterine fibroid embolisation using MRI perfusion parameters and patient questionnaires: preliminary results

**DOI:** 10.1186/s12880-022-00926-y

**Published:** 2022-12-05

**Authors:** Maliha Sadick, Leonie Hofmann, Christel Weiß, Benjamin Tuschy, Stefan O. Schönberg, Frank G. Zöllner

**Affiliations:** 1 Clinic for Radiology and Nuclear Medicine, University Medical Centre Mannheim, Heidelberg University, Theodor-Kutzer-Ufer 1-3, 68167 Mannheim, Germany; 2 Department for Medical Statistics and Biomathematics, Medical Faculty Mannheim, Heidelberg University, Theodor-Kutzer-Ufer 1-3, 68167 Mannheim, Germany; 3 Department for Gynaecology and Obstetrics, University Medical Centre Mannheim, Heidelberg University, Theodor-Kutzer-Ufer 1-3, 68167 Mannheim, Germany; 4 Computer Assisted Clinical Medicine, Medical Faculty Mannheim, Heidelberg University, Theodor-Kutzer-Ufer 1-3, 68167 Mannheim, Germany; 5 Mannheim Institute for Intelligent Systems in Medicine, Medical Faculty Mannheim, Heidelberg University, Theodor-Kutzer-Ufer 1-3, 68167 Mannheim, Germany

**Keywords:** Uterine fibroid, Leiomyoma, Uterine fibroid embolisation, Uterine artery embolization, MRI-perfusion, Dysmenorrhea

## Abstract

**Background:**

Uterine fibroid embolisation (UFE) is an established treatment method for symptomatic uterine myomas. This study evaluates the efficacy of UFE using objective magnetic resonance imaging (MRI) data for size and perfusion analysis as well as patient questionnaires assessing fibroid-related symptoms.

**Method:**

Patients underwent MR-Angiography before UFE and 4 days, 6 and 12 months after the procedure. The images were evaluated using dedicated software. Patient questionnaires were completed before UFE and at 12 months follow-up, focussing on the embolization procedure and symptoms associated with uterine fibroids. Statistical analysis of the questionnaires was performed using paired sample t-test and Wilcoxon signed rank test, while Kruskal–Wallis test and Friedman test were applied for MRI-analysis.

**Results:**

Eleven women were included. There was a significant reduction in fibroid-related symptoms. The volume reduction after 12 months was significant in both, uterus and myomas, after an initial increase in uterine volume at the first post-interventional MRI. The perfusion analysis showed that blood flow to the fibroids could be significantly reduced up to 12 months after UFE while uterine tissue was not affected.

**Conclusion:**

This study shows that uterine fibroid embolisation induces a significant long-term decrease in myoma size and perfusion while healthy uterine tissue remains unaffected. Fibroid-related symptoms are reduced for the sake of improved quality of life.

## Background

Uterine fibroids are benign neoplasms of the uterus that are made up of smooth muscle cells and occur in up to 70% of white women and 80% of women of African descent by the age of 50 years [1, 2]. High estimated direct and indirect costs underline a substantial socioeconomic impact [3]. The growth of fibroids is linked to the hormonal activity of the ovaries [4]. Accordingly, many fibroids regress spontaneously after menopause [5]. The causes of fibroids have not been conclusively clarified. However, it is assumed to be a multifactorial process involving hormonal dysregulation and genetic factors [6, 7]. Myomas can be classified according to their location, ranging from subserosal to submucosal [7].

Approximately 15–30% of women with uterine fibroids experience symptoms with potential limitations of the patient's quality of life [5, 8, 9]. The most common symptoms include bleeding disorders such as hyper- and dysmenorrhoea, as well as meno-/ metrorrhagia and even anaemia, micturition and defecation difficulties, dyspareunia and recurrent miscarriages [10, 11].

Myomas are mainly diagnosed when symptoms such as bleeding disorders occur. Myoma-induced hypermenorrhoea must be weighed against endometriosis or malignancies [12, 13]. Transvaginal ultrasound is the most commonly available diagnostic tool whereas magnetic resonance imaging (MRI) is used for intervention planning [13].

Symptomatic myomas should be treated [14, 15]. The choice of treatment method requires individual consideration. The spectrum includes hormonal therapy, minimally invasive procedures, and surgery. Each method has its challenges and advantages [16, 17].

Uterine fibroid embolisation (UFE) represents one of the most established minimally invasive procedures for the treatment of symptomatic uterine fibroids, first described by Ravina et al. in 1995 [18, 19]. This study evaluates its outcome based on perfusion parameters in MRI, before and up to 12 months after the intervention. Furthermore, changes in clinical symptoms of the women undergoing UFE were monitored by means of pre- and post-interventional patient questionnaires.

## Materials and methods

### Study design

This study was conducted prospectively using MRI examinations before and up to 12 months after uterine fibroid embolisation. Changes in size and perfusion parameters of treated fibroids were recorded and pre- and post-interventional patient questionnaires monitoring changes in the patients’ clinical symptoms were analysed. This prospective approach was approved by the hospital's Ethical Committee and in all patients written informed consent for MRI, UFE and follow-up, including questionnaire assessment, was obtained. All UFEs were performed by the same interventionalist using a standardised embolic agent, approved for UFE treatment, composed of Tris-Acryl Gelatine Microspheres (TAGM). All participants were examined by MRI according to a standardised study protocol at the following four time points: Before embolisation (pre), 4 days after embolisation (post) as well as 6 and 12 months after the intervention (6 month follow-up and 12 month follow-up). Additionally, all patients filled out a pre-interventional questionnaire which was repeated 12 months after UFE to evaluate the patients` clinical condition and quality of life.

### MR imaging

The standardised imaging protocol at a 3T scanner (MAGNETOM Skyra, Siemens Healthineers Inc., Erlangen, Germany) comprised T1 and T2 weighted turbo spin echo (TSE) sequences, a T2 weighted turbo inversion recovery magnitude (TIRM) sequence for morphological assessment of the lesions and a time-resolved angiography with interleaved stochastic trajectories (TWIST) sequence for contrast-enhanced MR perfusion measurements. Sequence parameters are summarised in Table 1. Morphological imaging was performed in two slice orientations and w/o fat saturation.

**Table 1:** MRI protocol for all examinations of the pelvis

Sequence	Plane	TR [ms]	TE [ms]	Flip angle	Slice thickness	Inter-section gap	Slices	Matrix size	Acquisition time [min]
T2_tirm	Cor	2700	45	160°	7 mm	1,4	27	288 × 384	1:06
T2_tirm	Axial	2700	43	160°	7 mm	1,4	27	168 × 320	0:39
T2_tse	Cor	2300	108	160°	6 mm	1,2	15	307 × 384	1:20
T2_tse	Axial	3100	108	160°	6 mm	1,2	21	261 × 384	1:47
T2_tse_fs	Sag	3200	108	160°	6 mm	1,2	21	288 × 384	1:38
T1_tse_fs	Axial	629	11	150°	6 mm	1,2	21	203 × 320	2:04
TWIST_ iso	Cor	2,7	0,98	25°	1,6 mm	N/A	104	256 × 256	3:00
T1_tse_fs	Cor	463	9,3	150°	6 mm	1,2	15	240 × 320	1:13
T1_tse_fs	Axial	629	11	150°	6 mm	1,2	21	203 × 320	2:04
T1_tse_fs	Sag	634	9,3	150°	6 mm	1,2	21	224 × 320	1:32

*Cor* Coronal, *Sag* Sagittal, *fs* Fat saturation, *TE* Echo time, *TR* Repetition time

TWIST view sharing was set to outer/ inner sampling density of 15/20% as described by Sadick et al. [20] Using parallel imaging (GRAPPA, PAT 3), a nominal temporal resolution of 1.5 s was achieved. A solution consisting of 0.1 mmol/kg body weight contrast agent (Dotarem, Guerbet, France) was administered intravenously after the acquisition of five baseline images, followed by a saline flush of 10 ml. In total 88 volumes were recorded. T1 weighted contrast-enhanced multiplanar sequences followed.

### Image analysis

The volume of uterus and fibroids was measured in the T1-weighted transversal images by manually tracing their outlines segmentally, using the volumetry tool of the software Aycan Workstation Pro (Version 3.14.006, Aycan Medical Systems LLC, Rochester, New York).

Perfusion analysis was performed as previously reported [20]. Briefly, maps of blood flow (BF), blood volume (BV) and mean transit time (MTT) were calculated by a voxel-by-voxel deconvolution approach using an in-house certified plugin (UMMPerfusion 1.5.3) [21, 22]. The arterial input function was selected by placing a region-of-interest (ROI) in the aorta proximal to the bifurcation. All data were normalised by subtracting the mean intensity of 5 baseline volumes and a linear relationship between contrast agent concentration and signal intensities was assumed due to the low dose of injected contrast agent.

A maximum of three fibroids per patient were selected for volume and perfusion analysis. Myoma selection criteria included size in descending order, clear delineation from surrounding uterine tissue and therefore optimal size and volumetric assessment [20]. The anatomical position was recorded to keep track of each myoma over time.

### Patient questionnaire

The patient questionnaire, created for this study, in consensus between gynaecologist and interventional radiologist, included 56 items. First, demographic data such as age, height and weight were collected, followed by questions about the patients’ expectations towards UFE therapy and the individual history of the disease. This included the duration of symptoms and the number of doctors previously consulted in connection with symptomatic uterine fibroids as well as previous therapeutic attempts. The second section of the questionnaire was omitted in the post-interventional survey, as the questions referred specifically to the pre-interventional condition. Both versions of the questionnaire included general myoma-related symptoms such as bleeding disorders with 22 items in total and 9 questions on symptoms regarding sexual discomfort, such as dyspareunia, in connection with myomas.

### Statistical analysis

To assess possible significant differences in the obtained volumes and perfusion parameters, the Kruskal–Wallis test was employed to evaluate whether the group medians (pre, post and 6 month and 12 month follow up) differ. Due to high variabilities in the ROI volumes at baseline, for comparing the change in volume over time, the Friedman test was applied. Based on this, a post-hoc test for pairwise comparison of the group means was performed using the Bonferroni method to correct for multiple comparisons. A *p*-value of < 0.05 was considered significant. Statistical analysis was carried out using Matlab 2016a (The Mathworks, Natick, USA). Post-hoc power analysis for the given number of included patients was performed showing that all statistical tests had a power of at least 0.955 for already n = 7 [20]. This implies enough samples for this study.

For better summary and comparison of the patients´ symptoms, the individual items of the pre- and postinterventional questionnaire were combined into sum scores using the Wilcoxon signed rank test. The scores ranged from 0 to 100%, with 100% representing the maximal manifestation of fibroid-related symptoms. Individual symptoms were compared using paired sample t-tests.

## Results

### Demographic data and information pertaining to UFE

MRI data and questionnaires of 11 patients with an average age of 46 years (range 43–51 years) were evaluated. The average observation period from the first MRI and distribution of the pre-interventional questionnaire to the second follow-up MRI and return of the post-interventional questionnaire was 422 ± 70 days.

The desire to have children was denied by 8/11 patients (72.8%). The mean duration of myoma disease was 1–3 years (63.7%) during which patients were mainly affected by the physical strain caused by the fibroids (72.8%). 10/11 women (91%) had consulted 1–3 doctors before consenting to UFE and 9/11 patients (81.9%) had been informed about alternative treatment options for symptomatic fibroids. Most women were informed about two options only, hormonal therapy and hysterectomy (72.8%). UFE as a treatment modality had been presented to five women (45.5%), followed by myomectomy in four cases (36.4%) and HIFU in three patients (27.3%). Five patients had previously been treated with progesterone receptor modulators (45.5%). When deciding on UFE, organ preservation was crucial for the patients (91%), while aspects such as a short duration of hospitalisation (36.4%) and relatively low expected pain levels (27.3%) were considered less important. Accordingly, most women expected preservation of the uterus (72.8%) and control of hypermenorrhoea (72.8%) after UFE. The least important aspect for the patients was the fulfilment of an existing desire to have children (27.3%). The pain expected to be associated with UFE was considered mild (54.6%) or moderate (18.2%) and the risks associated with the intervention were assessed as non-existent (27.3%) or very low (54.6%).

### Fibroid-related symptoms

The calculated symptom scores were 48% for general fibroid-related symptoms before the intervention and 24.09% 12 months later, resulting in a reduction of 23.91% which was significant (*p* = 0.042). The query on sexual symptoms resulted in a score of 14.91% before UFE and 11.64% afterwards, with a non-significant reduction of 3.27% (*p* = 0.195). There was a significant reduction in several individual symptoms such as the impairment of “general sense of well-being” (*p* = 0.025), hypermenorrhoea (*p* = 0.038), dysmenorrhoea (*p* = 0.01) and irregular duration of the menstruation (*p* = 0.016). Table 2 depicts the scores of these symptoms for each patients at time points pre intervention and after 12 months.

**Table 2:**
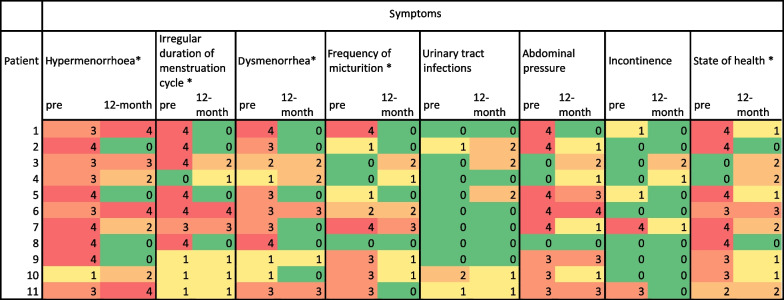
Patient individual scored symptoms before the intervention and at 12 month follow-up

Scores ranged from 0: no symptoms to 4: extensive symptoms. Symptoms were color coded from green (0) to red (4) to display changes between pre and 12 month follow-up visually. Column headings denoted by *, depict significant changes. All data obtained by the same questionnaire handed out to the patients at the respective time points

### MRI analysis

**Fig. 1:**
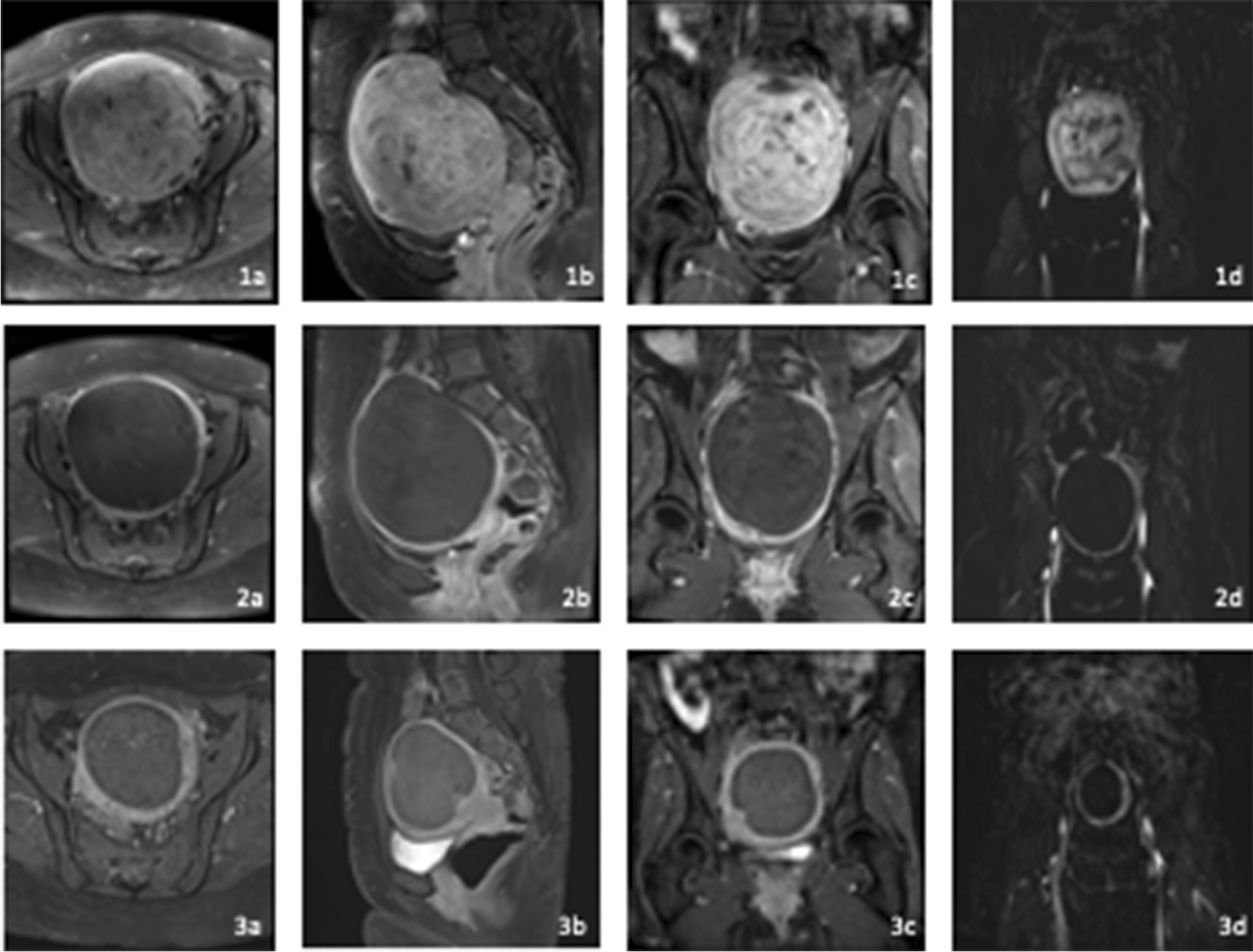
Contrast-enhanced MRI of an extensive uterine fibroid in a symptomatic patient pre (1), post (2) and at 12 month follow-up after UFE (3) **a** T1 axial, **b** T1 sagittal, **c** T1 coronal, **d** perfusion map in coronal view (blood flow)

**Fig. 2:**
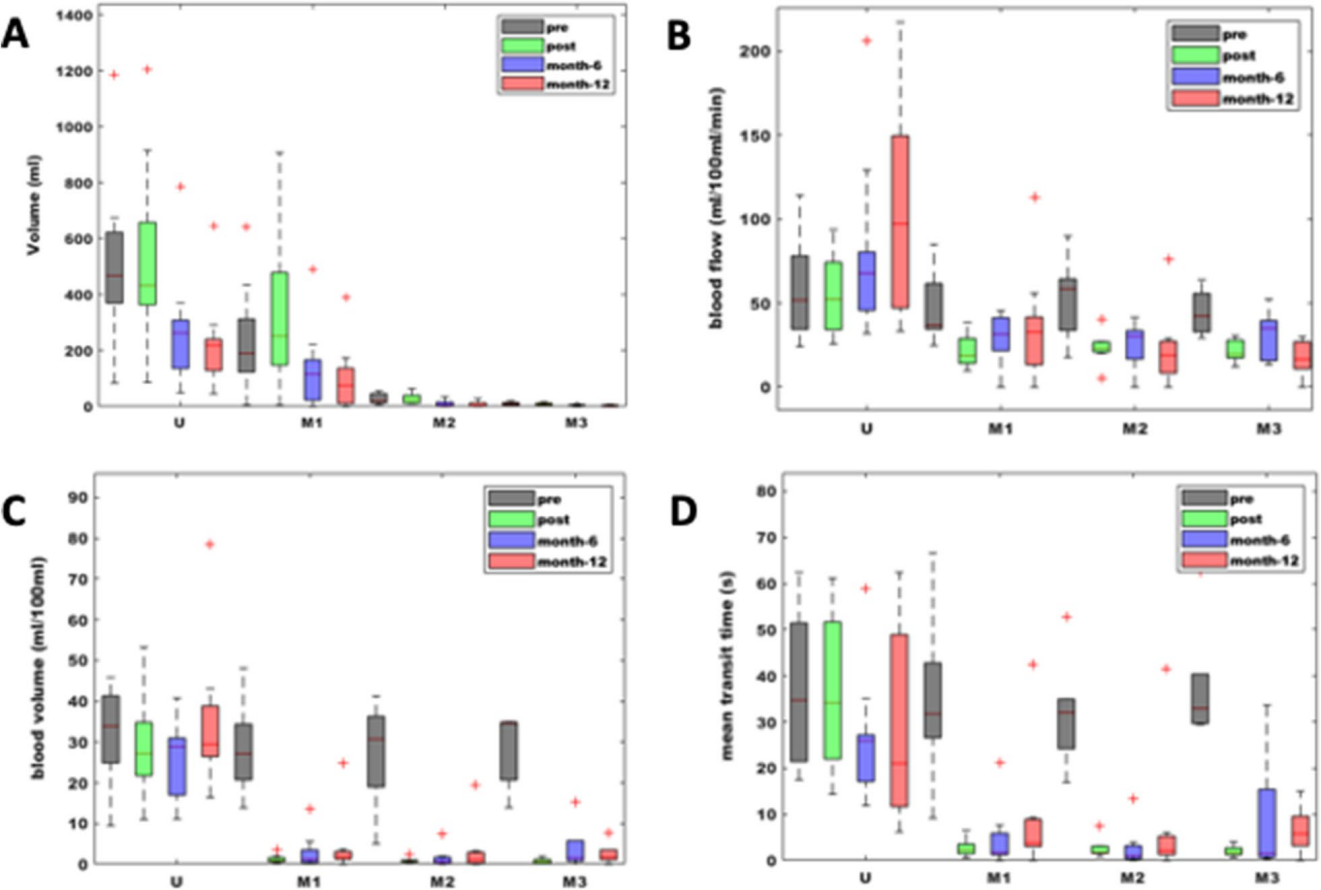
Boxplots of the **a** volume, **b** BF, **c** BV and **d** MTT for the uterus tissue and myomas according to pre, post, 6 month, and at 12 month follow-up after UFE. The median of the data is depicted by the line in the box while the 25% and 75% quartiles

Each patient underwent MRI before and 4 days after UFE. The first follow-up MRI was performed 206 ± 46 days and the second 407 ± 70 days after UFE. Figure 1 depicts exemplarily contrast-enhanced MRIs of an extensive uterine fibroid in a symptomatic patient pre, post, and at 12 month follow-up after UFE. The changes in uterine and fibroid volume and perfusion and the corresponding p-values are displayed in Table 3; Fig. 2.

**Table 3:** Measured volume and perfusion parameters given as mean and standard deviation in all patients for representative ROIs of healthy uterus and myoma tissue at the four selected time slots “Pre, Post, 6 month follow-up and 12 month follow-up”

Tissue	Parameter	Pre	Post	6 month	12 month	*p*-values
Uterus	Volume (ml)	498 ± 291	510 ± 324	272 ± 200	224 ± 159	0.0068
	BF (ml/min/100ml)	60 ± 30	53 ± 23	78 ± 50	106 ± 65	0.1226
	BV (ml/100ml)	32 ± 11	29 ± 12	25 ± 10	35 ± 16	0.3617
	MTT (sec)	37 ± 16	36 ± 17	26 ± 13	30 ± 20	0.397
Myoma 1	Volume (ml)	234 ± 138	321 ± 279	128 ± 141	95 ± 115	0.0255
	BF (ml/min/100ml)	47 ± 21	21 ± 9	30 ± 14	35 ± 31	0.0096
	BV (ml/100ml)	29 ± 10	1 ± 1	3 ± 4	4 ± 7	< 0.0001
	MTT (sec)	36 ± 17	3 ± 2	4 ± 6	8 ± 12	< 0.0001
Myoma 2	Volume (ml)	29 ± 19	29 ± 20	11 ± 13	8 ± 11	0.0129
	BF (ml/100ml/min)	53 ± 24	24 ± 10	26 ± 14	24 ± 25	0.0661
	BV (ml/100ml)	27 ± 13	1 ± 1	2 ± 3	4 ± 7	0.0026
	MTT (sec)	32 ± 12	3 ± 2	3 ± 5	8 ± 15	0.0028
Myoma 3	Volume (ml)	11 ± 7	10 ± 5	4 ± 3	3 ± 3	0.0366
	BF (ml/100ml/min)	45 ± 14	22 ± 7	30 ± 16	17 ± 12	0.045
	BV (ml/100ml)	28 ± 10	1 ± 1	4 ± 6	3 ± 3	0.0104
	MTT (sec)	38 ± 14	2 ± 1	9 ± 14	7 ± 6	0.0251

*BF* Blood flow, *BV* Blood volume, *MTT* Mean transit time

The largest fibroid volume in each patient was grouped as “myoma 1” and measured 234 ± 138 ml before embolisation. In four patients (36.4%) the fibroids were so extensive that they formed a conglomerate, which in these cases, was also assigned to group “myoma 1”, as individual fibroids could no longer be distinguished. In seven patients (63.7%) a second myoma could be differentiated and in five patients (45.5%) a third one, which were summarised as groups “myoma 2” and “myoma 3”. The reduction in total uterine volume was significant (*p* = 0.007) after 12 months. The reduction in myoma volume was also significant with *p*-values of 0.026 (myoma 1), 0.013 (myoma 2) and 0.037 (myoma 3). Even when all fibroids were combined as a single sample, there was an overall significant volume reduction (*p* = 0.005). This volume reduction of fibroids and uterus was only significant at the first follow-up exam. Comparing the two time points “pre” and “post”, the volume of both, uterus and fibroids, increased slightly. A reduction in volume was then seen in the comparison from “post” to “6 month follow up”, which was, however, only significant at “12 month follow-up” (*p* = 0.033 for uterine tissue and *p* = 0.024 for all fibroids combined). Uterine tissue perfusion values were not significantly reduced over the 12 month period (p(*BF*) = 0.123, p(B*V*) = 0.362 and p(*MTT*) = 0.397). In the groups “myoma 1” and “myoma 3”, the three perfusion parameters BF, BV, and MTT decreased significantly over the 12 month observation period (see Table 3). The reduction in BF in the group “myoma 2” was not significant (*p* = 0.066), while BV (*p* = 0.003) and MTT (*p* = 0.003) decreased significantly. When all myomas were included into a single sample (*myoma *1–3) and the perfusion values measured at selected time points were compared, it could be seen that all perfusion values were significantly reduced in the pairwise comparison from the time point “*pre*” to the time points “post”, “6 month follow up” and “12 month follow-up” respectively (see Table 4). No significant changes were shown in the pairwise comparisons of the time points after UFE (see Table 4).

**Table 4:** p-values of multiple pairwise comparisons of perfusion parameters for the four selected time points “Pre, Post, 6 month follow-up and 12 month follow-up”

Parameter	Tissue	Pre/post	Pre/6 month	Post/6 month	Pre/12 month	Post/12 month	6 month/12 month
Volume	Uterus	1	0.1453	0.1614	0.0285	0.0329	0.9165
	M1-M3	1	0.2095	0.1477	0.0364	0.0237	1
BF	Uterus	0.9907	0.8322	0.6614	0.2386	0.1304	0.7324
	M1-M3	< 0.0001	0.0249	0.4155	0.0003	1	1
BV	Uterus	0.8239	0.4718	0.9379	0.9998	0.7799	0.4212
	M1-M3	< 0.0001	< 0.0001	1	< 0.0001	0.7284	1
MTT	Uterus	1	0.5556	0.5769	0.6613	0.6820	0.9984
	M1-M3	< 0.0001	< 0.0001	1	< 0.0001	1	0.8970

*BF* Blood flow, *BV* Blood volume, *MTT* Mean transit time, *M1-M3* Myoma tissue, all data grouped together

## Discussion

This study, based on morphological and functional imaging of symptomatic uterine fibroids, shows a decrease in clinical symptoms in patients after UFE as well as a significant reduction of fibroid tissue volume and perfusion up to 12 months, while the perfusion of the uterine tissue is not affected. This is in accordance with previous findings using a similar approach [20].

Most of the women in this study group, despite having consulted 1–3 gynaecologists, had only been informed about selective progesterone receptor modulators and hysterectomy as therapeutic options. This may be related to limited exposure to minimally invasive treatment options such as UFE, which should be performed in dedicated centres with on campus availability of interventional radiologists. Clements et al. [23] similarly noticed a low uptake of UFE in Australia and encouraged further exchange between interventional radiologists and gynaecologists in order to broaden the treatment spectrum for symptomatic fibroids. Although hysterectomy is the only definitive therapy for uterine fibroids, more and more women desire organ preservation and therefore choose UFE as an organ-sparing method [24, 25]. Very few patients only mentioned the wish for potential pregnancy after UFE. This is related to the average age of 46 years in our patients and the fact that UFE was not considered as first line therapy in women wishing to have children. Pregnancy after UFE is currently being discussed with heterogenous findings. Recent studies by Ludwig et al. and Ghanaati et al. however indicate that pregnancy is possible and may not involve higher risks than pregnancy without UFE [26, 27].

The evaluation of the questionnaires showed a significant overall reduction in general fibroid-related symptoms while the reduction of sexual complaints caused by myomas was not significant. However, the initial level of sexual discomfort as stated by our patients was very low. This being a rather sensitive topic, patients may have been hesitant to give information about it. Kovacsik et al. [28] evaluated the effect of UFE on sexual function in 264 women and found that one year after UFE, sexual function and quality of life had significantly improved. Further studies that specifically analyse the effects of uterine fibroids on sex life with larger samples would be required to generate additional data.

The results of the MRI examinations show a significant reduction in fibroid volume and perfusion. Consequently, the total volume of the uterus was also significantly reduced which underlines the successful outcome. The first post-interventional MRI showed a slight increase in the measured uterine volumes. This may be caused by the inflammatory reaction triggered by UFE, resulting in initial perifocal oedema and local swelling of the tissue. However, since a significant reduction of all volume values could be shown in the long-term evaluation, it can be assumed that this is a short-term reaction that does not limit the therapeutic success. In addition, no significant perfusion reduction was observed in healthy uterine tissue. On the contrary, the mean values for BF and BV increased. This shows that UFE achieves targeted embolisation of small arteries supplying the fibroids while healthy uterine tissue is preserved. The treated fibroids showed a significant reduction in the measured perfusion parameters for all time points after treatment.

The main limitation of the study is the sample size of only 11 women. Our intention was to utilize MRI as a standardized and objective follow-up tool in women after myoma embolization. The method is expensive and not commonly available, but despite a small sample size, we could establish a proof of principle and demonstrate that MRI imaging is an objective method for preinterventional but also postinterventional management of patients after UFE. It was essential for us to demonstrate these preliminary results as early as possible in order to prevent mismanagement of women in the outpatient setting after embolization. In cases where follow up of patients had been entirely based on ultrasound information by the referring gynaecologist, unfortunately many of our patients were told that the myoma load was still present after UFE. MRI information in these patients was essential for an objective diagnosis. This procedure and the interdisciplinary management of patients between gynaecology and interventional radiology is not commonly available after all and only possible in specialised centres. However, the results in this study are unambiguous and in line with findings in literature where studies have been performed with larger samples.

## Conclusion

Uterine fibroid embolisation achieves both, significant symptom relief and a long-term reduction in fibroid and uterine volume, as well as a reduction in perfusion of the treated fibroid tissue. Healthy uterine tissue remains unaffected which underlines the safety and efficacy of this treatment method in experienced centres. It can therefore be recommended to women with symptomatic uterine fibroids, even if extensive in size.

## Data Availability

The data presented in this study are available on request from the corresponding author. The data are not publicly available due to data protection and patient privacy regulations.

## Abbreviations

BF: Blood flow
BV: Blood volume
MTT: Men transit time
UFE: Uterine fibroid embolisation
